# Diagnostic Circulating miRNAs in Sporadic Amyotrophic Lateral Sclerosis

**DOI:** 10.3389/fmed.2022.861960

**Published:** 2022-05-06

**Authors:** A. Panio, C. Cava, S. D’Antona, G. Bertoli, D. Porro

**Affiliations:** Institute of Molecular Bioimaging and Physiology, National Research Council (IBFM-CNR), Milan, Italy

**Keywords:** sporadic amyotrophic lateral sclerosis, sALS, diagnosis, microRNA, circulating biomarkers

## Abstract

Amyotrophic Lateral Sclerosis (ALS) is a fatal neurodegenerative disease characterized by the neurodegeneration of motoneurons. About 10% of ALS is hereditary and involves mutation in 25 different genes, while 90% of the cases are sporadic forms of ALS (sALS). The diagnosis of ALS includes the detection of early symptoms and, as disease progresses, muscle twitching and then atrophy spreads from hands to other parts of the body. The disease causes high disability and has a high mortality rate; moreover, the therapeutic approaches for the pathology are not effective. miRNAs are small non-coding RNAs, whose activity has a major impact on the expression levels of coding mRNA. The literature identifies several miRNAs with diagnostic abilities on sALS, but a unique diagnostic profile is not defined. As miRNAs could be secreted, the identification of specific blood miRNAs with diagnostic ability for sALS could be helpful in the identification of the patients. In the view of personalized medicine, we performed a meta-analysis of the literature in order to select specific circulating miRNAs with diagnostic properties and, by bioinformatics approaches, we identified a panel of 10 miRNAs (miR-193b, miR-3911, miR-139-5p, miR-193b-1, miR-338-5p, miR-3911-1, miR-455-3p, miR-4687-5p, miR-4745-5p, and miR-4763-3p) able to classify sALS patients by blood analysis. Among them, the analysis of expression levels of the couple of blood miR-193b/miR-4745-5p could be translated in clinical practice for the diagnosis of sALS.

## Introduction

Amyotrophic Lateral Sclerosis (ALS) is a fatal neurodegenerative disease characterized by the neurodegeneration of motoneurons in the cortex, brainstem and spinal cord. The upper motoneurons in the brain degenerate and become unable to communicate to those of the spinal cord, which in turn don’t send any message to the muscles. Unstimulated muscles weaken during time, start to twitch and become atrophic (1). ALS is considered an “orphan disease” affecting 1–2/100,000 with a total of about 5/100,000 people per year around the world (2).

The disease causes high disability and a high mortality rate, and nowadays there is no effective treatment to control disease onset and progression (3).

Nearly all cases of ALS are considered sporadic, with no clear association to risk factors, environmental factors (4) or family history, as only 5–10% of the cases are hereditary from the family, due to a gene mutation (5). Several of the proteins, associated with hereditary ALS, are involved in microRNA (miRNA) processing (6–10). Another cause possibly linked to ALS is the accumulation of proteins, due to altered protein clearance (11). Finally, a mutation in the lipid metabolism *SPTLC1* gene has been found in only one ALS family (12).

The actual diagnosis of both hereditary and sporadic forms of ALS (sALS) is based on EI Escorial criteria (13), clinical assessment, electrophysiological examination and exclusion of other pathologies mimicking ALS. Hereditary ALS (or familial ALS, fALS) diagnosis is also based on the genetic screening for familial cases, while the lack of diagnostic tools for sporadic cases hampers the early disease diagnosis and delays the treatment of the pathology. Although the pathogenesis of sALS is largely unknown, analyzing the animal models of genetic ALS (*C9orf72*, copper/zinc superoxide dismutase 1 (SOD1) and TAR DNA-binding protein 43 mouse mutants (14) there is emerging evidence that several distinct molecular mechanisms may play a role (oxidative stress, glutamate excitotoxicity, protein misfolding, …) (15). The development of new clinical biomarkers for early ALS detection, for the subclassification of the cases on the base of the phenotype and for the addressing to the best therapeutic option are urgently required. Furthermore, there is no diagnostic test for sALS and the diagnosis is based on the onset of symptoms and the disease course. In this rapidly progressive disease, there is also a lack of potential effective strategies, as well as disease biomarkers to classify the various degree of pathology progression.

**FIGURE 1:**
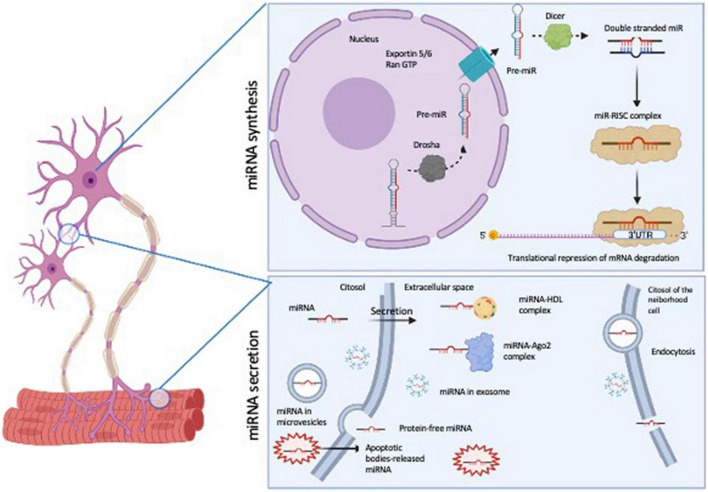
Schematic representation of miRNA biosynthesis and secretion pathways. The figure was generated by BioRender software (BioRender.com).

MicroRNAs (miRNAs) are emerging as important molecules involved in ALS pathogenesis (16). miRNAs are defined as 21–25 nucleotide, non-coding, single-stranded RNAs (ssRNA), which are derived from larger precursors that form stem-loop structures. miRNAs are processed into a primary miRNA (pri-miRNA) by Drosha; the pri-miRNA is released in the cytoplasm by Exportin 5/RanGTP, where it is handled by Dicer to create a miRNA duplex. The mature miRNA binds to Ago 1/2 and with other proteins to form miRNA-induced silencing complex (miRISC), the site where the miRNA precisely recognizes and binds the mRNA target usually in its 3′untranslated region (3′ UTR) in order to regulate the mRNA expression (Figure 1 upper panel).

MicroRNA function is inserted in a complex regulatory network: hundreds of protein-coding mRNAs are regulated by miRNAs, and a single miRNA could regulate several target mRNAs (17). Nevertheless, miRNAs are tissue specific, and their expression profile and function in the epigenetic control of gene expression are specific for different pathologies. These features make them interesting molecules in the research field of new biomarkers of pathology. Another important characteristic of miRNA molecules is that they are resistant to freeze-thaw cycles (18), and they can be found in circulating biofluids (saliva, lacrimae, urine, cerebrospinal fluid- CSF- and blood); they are also extremely stable also at low pH or harsh condition, suggesting that they are resistant to extracellular RNAse activity (18). Their profiling could give an overview of their expression levels in normal and pathological conditions by microarray, RNA sequencing (RNA-seq) and Real Time quantitative PCR (RT-qPCR) techniques (2). The mechanisms of secretion could be mediated by their capability to associate to either proteins (AGO2) or to be included in lipoprotein complexes with HDL or LDL, that allow them to be protected from degradation [for a review see (19)]. Other studies have demonstrated that miRNAs are also wrapped with membrane vesicles (exosome, microvesicles and apoptotic bodies), but it is also possible to find free-circulating miRNAs, which are the bulk of extracellular miRNAs (20) (Figure 1, lower panel). On the role of extracellular miRNA, the main hypothesis is that their release in biofluids would help the cell-cell communication, suggesting the transport of these molecules also far from the tissue of origin. Moreover, exosome wrapped miRNAs can alter gene expression in recipient cells, confirming the possibility of extracellular miRNAs to be important tools for intracellular communication.

The best ideal biomarker should be easy to be isolated and analyzed, low- and time-efficient, and, possibly, minimally invasive for the ALS patients. In this context miRNAs are emerging as interesting molecules also for ALS, being tissue-specific, strongly correlated with the pathology and minimally invasive to be isolated and measured, if isolated in human biofluids. Indeed, the use of human fluids as starting materials for miRNA expression analyses could be easier and acceptable by the patients than the collection of bioptic tissue samples. The possibility to analyze miRNA expression levels in biofluids makes them interesting candidates to become ALS biomarkers, in particular in the sporadic forms. Indeed, several studies are describing the potential use of circulating miRNAs as biomarkers for sporadic ALS (sALS) diagnosis, although a specific, unique panel of miRNAs associated with sALS is not present. The goal of this analysis is to give an overview of the possible use of miRNAs as diagnostic molecules in sALS.

## Materials and Methods

### miRNA Identification in Literature

**FIGURE 2:**
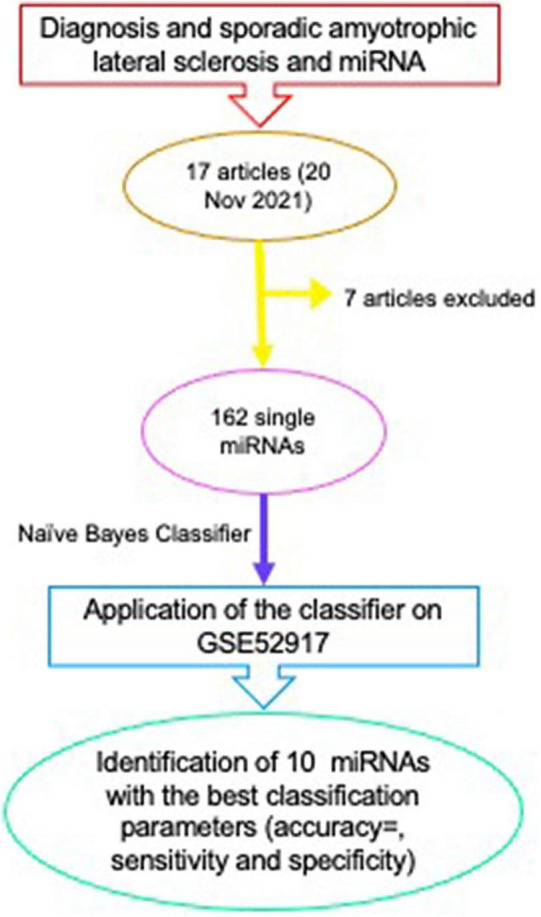
Scheme of the method for identification of a panel of miRNAs with diagnostic properties in sALS.

With the aim of identifying a single miRNA or a miRNA profile with diagnostic ability in sALS, we collected all the papers listed on PubMed, searching for “diagnosis and sporadic amyotrophic lateral sclerosis and miRNA” (Figure 2). The query revealed the publication of 17 articles (20 November 2021); from all these articles all those regarding genetic forms of fALS (21); those describing genetic animal models (22, 23); those not specifically related to sALS (24), all the reviews on diagnostic miRNAs in genetic and sporadic forms of ALS (16) and those non-accessible (25, 26) were excluded.

The methods used in all these articles to obtain sALS-associated miRNA profiles include microarray analyses, next generation sequencing (NGS), but also the analysis of a single miRNA by RT-qPCR in different populations, comparing healthy subjects or patients with other neurodegenerative pathologies to sALS patients. miRNAs have been isolated from either spinal cord tissues or from blood, serum or plasma samples.

### Bioinformatics Approach for miRNA Classification

In order to assess the diagnostic role of literature-identified miRNAs, we implemented a Naive Bayes classifier (27), using an R Bioconductor package, e1071 (28). Our classifier was applied on an ALS dataset downloaded from the Gene Expression Omnibus (GEO), GSE52917 (29). It contains miRNA expression levels extracted from human serum and we selected the expression levels of 18 sALS patients and 17 controls. The aim of Naïve Bayes classifier was to develop: (i) a predictive model which is able to learn how to distinguish sALS patients and controls in the training set, and (ii) use the generated predictive model to classify new samples in sALS or control in the testing set.

First, we normalized the expression levels of miRNAs performing the Min-Max Normalization method (30). Then, we divided the original data into a training (70%) and testing set (30%). The training set is represented by a matrix of samples belonging to sALS patients and controls (known classes) and selected miRNA expression levels for each sample. The testing set is composed of a matrix of samples of unknown classes with miRNA expression levels.

In order to verify the efficiency of our classification, we evaluated three parameters: accuracy, sensitivity, specificity in the testing dataset. Accuracy is defined as the number of correctly classified samples divided by the total number of classified samples. Sensitivity and specificity indicate the ability of the classifier to correctly classify the samples with and without a disease, respectively.

## Results

### Tissue Diagnostic miRNAs in Sporadic Amyotrophic Lateral Sclerosis

Microarray analysis of spinal cord samples of five sporadic ALS and five control patients with diseases other than neurodegenerative disorders revealed upregulation in 37 miRNAs, and the downregulation in 35 miRNAs (31). Further validation revealed a significant downregulation of miR-139-5p and a significant upregulation of miR-5572 in sALS. The prediction by TargetScan analysis of miR-5572 targets and the *in vitro* validation revealed that zinc transporters could be controlled by this miRNA. Zinc could be important as a cofactor for several biological activities.

The transcriptome deep sequencing of spinal cord ventral horns of post-mortem sALS human donors showed 1160 deregulated mRNAs and 18 miRNAs. The snapshot offered by transcriptomic profile revealed the downregulation of genes involved in relevant neurological functions (impulse transmission, synaptic transmission, calcium ion transport or neurotransmitter secretion) with a specific reduction of *SNAP25* and *STX1B* gene expression, neuronal *t-SNARE* involved in vesicle trafficking and calcium dynamics. miRNA seq analysis revealed miR-124-3p, -127-3p, -133a, -136-3p, -182-5p, -183,5p, -218-5p, -219-5p, -409-3p, -410, -478b, -485-5p, -577, -873-5p, -889, to be downregulated, while miR-148a-3p, -155-5p and -221-3p to be upregulated in these tissues. Several of the decreased miRNAs are involved in TGFβ protein signaling, and in the control of phosphatidylinositol-3 kinase (*PI3K*)/*AKT* pro-survival pathway (32). Alteration in the level of miR-219-5p, found in human spinal cord by RNA-seq by D’Erchia et al. (32), was also observed in *SOD1^G93A^* mice spinal cords possibly associated to gliosis (33).

### Circulating Diagnostic miRNAs in Sporadic Amyotrophic Lateral Sclerosis

In the first paper (34), the authors analyzed the expression of a single specific miRNA, miR-338-3p, in multiple samples, including blood leukocytes, serum, CSF, and spinal cord of sALS (*n* = 72 patients) compared to healthy controls (*n* = 62). In all these samples, this miRNA is overexpressed. The main potential target could be *SLC1A2* (transporter that clears glutamate) and host gene apoptosis-associated tyrosine kinase (*AATK*), which is overexpressed during apoptosis of myeloid precursor cells by IL3 deprivation. The last process would link miR-338 to apoptosis of mature neurons observed in sALS.

The microarray analyses of 18 sALS sera compared to 16 matched healthy control sera revealed the downregulation of 11 miRNAs (35). Only two miRNAs were validated, miR-1234-3p and miR-1825. Of these two miRNAs, miR-1234 is able to distinguish sALS from fALS. Regarding their regulatory function, the functional network “Neurological Disease, Skeletal and Muscular Disorders, Cell-To-Cell Signaling and Interaction” seems to be the target of these two miRNAs, with *NHPH3* and *NLE1* being the two most significantly predicted common targets of the two miRNAs.

With a microarray-based approach, in Takahashi et al. (36) the authors characterized plasma miRNA profiles of 16 sALS patients and 10 healthy controls. The majority of miRNA were downregulated, and in the validation phase only miR-663-b, miR-4258, and miR-4649-5p were upregulated in sALS, with hsa-miR-663b and hsa-miR-4649-5p showing a negative correlation with disease duration from onset to end point (for details see Table 1). Regarding the pathways regulated by these miRNAs, also these authors highlighted that ALS disease may involve alteration in the synthesis and maturing processing of miRNAs, neuronal development and neurogenesis, transmission of nerve impulse (miR-663b), and cell proliferation, mitosis and protein folding (let-7f-5p). Some of these miRNAs (miR-4299 and miR-4649-5p) could be involved in cell adhesion, angiogenesis and axonal guidance, all processes involved in the sALS onset.

**TABLE 1:** Summary table of miRNA associated to sALS (NR = not reported).

Differentially expressed miRNAs	Validated miRNAs	Tissue	Identification method	No. of samples	Possible target genes	References
miR-5572 (UP) miR-139-5p (DOWN)		Spinal cord	Microarray	*n* = 5 sALS vs. 5 HC	Zinc transporter	(31)
miR-24-3p, -149-3p, -371a-5p, -393-5p, -1207-5p, -3619-3p -4298, -4484, -4505, -4688, -4700-5p, -4736, -4739 (UP) miR-150-3p, -634, -1268a, -2861, -3176, -3177-3p -3605-5p, -3911, -3940-3p, -4507, -4508, -4646-5p, -4674, -4687-5p, -4745-5p, -4788 (DOWN)		Blood extracellular vesicles	Microarray	*n* = 5 sALS vs. 5 HC	Synaptic vesicle docking and exocitosis, positive regulation of neurotransmitter transport and secretion and synaptic vesicle cycle	(44)
miR-1, -10b-5p, -153-3p, -224-3p/5p, -326, -338-3p, -877-3p, -1296-5p, -3194-3p, -4695-3p, -5684 (UP in sALS blood); miR–125a-3p, -143-3p, -144-5p, -190a-5p, -193a-5p, -199b-5p, -218-5p, -618, -338-5p, -542-5p, -4423-3p (DOWN in sALS blood) miR-27a-5p, 142-5p, -150-3p, -223-3p/5p, -342-3p, -431-5p, -642a-5p, -766-3p, -4433b-5p, -5684, -6503-3p, (UP in neuromuscular sALS junction) miR-9-3p, -34b-5p, -100-5p, -125b-2-3p, -129-2-3p, -135a-5p, -138-5p, -204-5p, -211-5p, -214-3p, -218-5p, -3182, (DOWN in neuromuscular sALS junction)	miR-223-3p, miR-338-3p, miR-342, and miR-326 (UP in neuromuscular junctions); miR-224-3p, miR-338-3p and miR 5684 (UP in blood of sALS)	Blood and neuromuscular junction	NGS	*n* = 45 sALS vs. 25 HC	Neurogenesis: HIF1A, BDFN1, SNCA, and VEGFA. Axon development: BDFN1, RHOB, VEGFA, and KALRN. Neurotransmitter uptake: SNAP25, SNCA	(40)
miR-124-3p, -127-3p -133a, -136-3p, -182-5p, -183,5p, -218-5p, -219-5p, -409-3p, -410, -478b, -485-5p, -577, -873-5p, -889, (DOWN); miR-148a-3p, -155-5p and -221-3p (UP)		Spinal cord ventral horns	RNA-seq	*n* = 11 sALS vs. *n* = 7 HC	Neuronal transmission (SLC1A2, CHAT, SLC12A5, HTR2C). Synapse function (SNPH, SYT4, SNAP25, STX1B). Calcium metabolism (GRIN1, GRIN2A, CACNA1G). Cholesterol Biosynthesis (HMGCR, HMGCS1, MSMO1, SQLE).	(32)
miR-15b-5p, -20a-3p, -106b-5p, -133a-3p, -133b, -134-5p, -135b-5p, -143-3p, -144-3p, -145-5p, -146b-3p, -190a-5p, -196b-5p, -206, -214-3p, -301a-3p, -331-3p, -335-5p, -374b-5p, -381-3p, -500a-3p, -518d-3p, -532-3p, -551b-3p, -744-5p. -let7d-5p.	miR-206, miR-143-3p (UP); miR-374b-5p (DOWN)	Serum samples	TaqMan low density array	*n* = 27 sALS vs. 25 HC or patients with other pathologies	NR	(38)
miR-34a, -100, -193b, -4485 (UP); miR -124, -183, -451, -3690, -3935, -4538, and -4701 (DOWN)	Signature proposed: hsa-miR-183, hsa-miR-193b, hsa-miR-451, and hsa-miR-3935	Blood Leukocytes	Microarray	*n* = 5 sALS vs. 5 HC	PI3K/AKT signaling pathway, MTOR signaling pathway, regulation of actin cytoskeleton, axon guidance, MAPK signaling, glioma and gap junction	(37)
let-7a-5p, let-7d-5p, let-7f-5p, let-7g-5p, let-7i-5p, miR-15a-5p, -15b-5p, -16-5p, -22-3p, -23a-3p, -26a-5p, -26b-5p, -27b-3p, -28-3p, -30c-5p, -30b-5p, -103a-3p, -106b-3p, -128-3p, -130b-3p, -144-5p, -148a-3p, -151a-5p, -182-5p, -183-5p, -186-5p -221-3p, 342-3p, -425-5p, -451a, -584-5 (all DOWN)	let-7a-5p, miR-15a-5p, -16-5p, -26a-5p, -27b-3p, -128-3p, -148a-3p, -148b-3p, - -151a-5p	Blood samples	NGS	*n* = 56 sALS vs. *n* = 20 HC	ABCG1, LGALS3, CTDSP1, BAX, ITGA5, PRKCD, OTUB1, ZYX, ARHGDIA, HDGF, HMGA1, PKM, RAB40C, MYO1F, UCP2, PINK1, and PHB	(46)
3 upregulated and 120 downregulated miRNAs.	miR-663-b, -4258, and -4649-5p (UP); let-7f-5p, miR-26b-5p, -3187-5p, -4299, -4419a, and -4496 (DOWN)	Plasma	Microarray	*n* = 16 sALS vs. 10 HC	Synthesis and maturing processing of miRNAs, neuronal development and neurogenesis, transmission of nerve impulse; cell proliferation, mitosis and protein folding	(36)
miR-455-3p, -940, -1228-3p, -1234-3p, -1825, -1915-3p, -3665, -4270, -4689, -4745-5p, -4763-3p (DOWN)	miR-1234-3p and miR-1825 (DOWN)	Serum	Microarray	*n* = 18 sALS vs. 16 HC	NHPH3 and NLE1	(35)
miR-338-3p	miR-338-3p	Blood leukocytes, serum, CSF, spinal cord	RT-qPCR	*n* = 72 sALS vs. *n* = 62 HC	SLC1A2 AATK	(34)

In the comparison of microarray profile of leukocytes from 5 Chinese sALS patients and 5 Chinese healthy controls, Chen et al. (37) found miR-34a, miR-100, miR-193b, miR-4485, significantly higher in sALS compared to healthy subjects, while miR-124, miR-183, miR-451, miR-3690, miR-3935, miR-4538, and miR-4701, were downregulated in the same comparison. The significant differences in the expression levels of four miRNAs (hsa-miR-183, hsa-miR-193b, hsa-miR-451, and hsa-miR-3935) were confirmed in RT-qPCR. The downregulation of miR-124 and the increase of miR-34a observed in microarray of sALS blood leukocytes (37) was also reported by Zhou et al. (33) in *SOD^G93A^* animal model. After ROC curve analysis the authors claimed that the levels of miR-183, miR-193b, miR-451, and miR-3935 could be proposed as useful biomarkers for the diagnosis of sALS. Gene Ontology and KEGG database revealed the involvement of these miRNAs in the control of *PI3K/AKT* signaling pathway, *MTOR* signaling pathway, regulation of actin cytoskeleton, axon guidance, *MAPK* signaling, glioma and gap junction.

In Waller et al. (38), the authors used miRNA profile to identify a diagnostic signature in sera of sALS (*n* = 27) compared to that of healthy subjects or to patients with disease not related to ALS (*n* = 25). Fourteen significant diagnostic miRNAs in the comparison between sALS and healthy subject were found (for the details see Table 1). The validation phase on a separate case-control cohort confirmed that miR-143-3p, miR-206, (upregulated) and miR-374b-5p (downregulated) are differentially expressed between sALS and healthy subjects. In *SOD1^G93A^* transgenic mouse model miR-206 expression, analyzed in murine spinal cord by RT-qPCR, was increased at the pre-onset of the disease (39) and this upregulation is similar to that found in sALS serum samples by Waller et al. (38). The overexpression of miR-143-3p increased during the development of the pathology, as well as the decrease of miR-374b-5p.

In the next generation sequencing (NGS) analysis performed on blood and neuromuscular junctions of 45 sALS versus 25 healthy subjects (40), the authors found 20 upregulated miRNAs and 22 downregulated miRNAs in blood, and 284 upregulated and 222 downregulated miRNAs in neuromuscular junctions of ALS compared to healthy subjects (Table 1). The validation analysis revealed that miR-223-3p, miR-326 and miR-338-3p were elevated in ALS neuromuscular junction compared to blood samples in 97% of the patients. Target prediction analyses revealed that these miRNAs regulate the brain-derived neurotrophic factor (*BDNF*) implicated in metabolic syndrome and neurodegenerative disease, but also the pathway of HIF1, including *HIF1α*, *VEGFA*, *EGLN3*, *TFRC*, and *IGF*. Some other targets are involved in neuronal compartment (*ACTB, KALRN, MATN2*, and *RHOB*), differentiation, morphogenesis and development.

miR-218 and miR-133a, decreased in blood and neuromuscular junctions of sALS patients (32, 38, 40) and miR-138 in spinal cords of sALS subject (32, 40), were found on the contrary upregulated in *SOD1^G93A^* transgenic mouse model (41), possibly suggesting that these miRNAs in sALS control different genes and pathways from familial ALS.

miR-125b, downregulated in neuromuscular junction of sALS patiens (40), was found upregulated in spinal cord of *SOD1^G93A^* mouse model (42). miR-9, downregulated in neuromuscular junction of sALS patiens (40), miR-124, downregulated in blood leukocytes (37) and in spinal cord (32) of sALS were also decrease in *SOD1^G93A^* whole spinal cord (43).

Blood extracellular vesicles (EV) miRNAs from five sALS and age-matched healthy controls were isolated. The microarray analysis revealed 13 upregulated and 17 downregulated miRNAs (44). Among the possible biological processes regulated by these miRNAs, there are synaptic vesicle docking and exocytosis, positive regulation of neurotransmitter transport and secretion and synaptic vesicle cycle, suggesting a main role of synaptic vesicle-mediated transport in ALS development.

In the book chapter (45), the authors described all the possible genetic and epigenetic mechanisms that could generate altered expression of miRNAs found in fALS. The authors’ hypothesis is that the altered miRNA profile is a consequence of a deregulation in RNA transcription regulatory mechanisms, implying that miRNAs *per se* are not involved in the onset of the fALS pathology. In particular, they highlighted the role of TDP-43 protein as a modulator of AGO2. The lack of TDP-43 observed in fALS subjects could affect miRNA biogenesis, decreasing the expression of several miRNAs (miR-132-3p/5p, miR-143-5p/3p, miR-574-3p). Also, the loss of Dicer has been described in spinal cord degeneration, suggesting that the biogenesis and the processing of miRNA is critical in ALS development. The authors described a good correlation in the miRNA profiles between animal models and human samples. For instance, miR-155 has been described upregulated both in fALS in human and in SOD1*^G93A^* animal model, perhaps for its regulatory role on TGFβf1, on the stimulation of proinflammatory macrophagic response and on increased secretion of cytokines. Similarly, miR-29 is highly expressed in humans and in the *SOD1* mouse model. In human samples, as well as in mouse models, there is an increased activation of microglia, possibly supported by overexpression of miR-22, miR-125b, miR-146b, miR-155, and miR-365, being miR-125b involved in TGFβ regulation and miR-365 in IL6 production in animal models. Nothing is reported on miRNA alterations in the sporadic form of ALS. That is why this book chapter has not been included in our analytical review.

A remarkable event in ALS is also the mitochondrial dysfunction (46) due to the influence of miRNA activity on mitochondrial gene expression regulation. Indeed, mitochondrial miRNAs (mito-miRs) are a class of miRNAs that regulate mitochondrial gene expression and function, such as oxidative stress, cell metabolism, chemoresistance, and apoptosis. It could be useful, considering the role of mitochondria in sALS development by identifying those miRNAs whose loss or increase could impact on mitochondrial functions. miR-22 and miR-26b, altered in human blood samples of sALS (46), were found altered also in the spinal cord and brainstem of *SOD1^G93A^* transgenic mice (33). In Catanesi et al. (46) miR-27a/b and miR-335-5p upregulation could modulate the mitochondrial dynamics and activates the pathway of caspase 3/7 in sALS. Similarly, the silencing of miR-151b is involved in the overexpression of *PINK1*, *UCP2*, and *PKM*, genes of neurodegeneration.

The analysis of sALS-associated miRNAs described in the literature is summarized in Table 1.

### *In silico* Identification of Circulating miRNAs With Diagnostic Properties

The analysis of the 10 articles in the literature on sALS identified 162 single miRNAs with potential diagnostic properties, coming from different profiles and tissues (spinal cord, CSF, blood, serum, plasma) although an agreement among publications on a single miRNA or a miRNA-panel is still lacking. For this reason, we proceeded with the bioinformatics analysis.

In order to assess the diagnostic role of 162 miRNAs presented in the first column of Table 1, we implemented a Naive Bayes classifier (27), using an R Bioconductor package, e1071 (28). The classification was performed on GSE52917, but as GSE52917 does not contain all 162 miRNAs the classification was performed considering the expression levels of 101 out of 162 miRNAs. The search criteria to identify GEO datasets for our analysis were the key words “sporadic Amyotrophic Lateral Sclerosis AND mirna AND serum” in Homo Sapiens (Access: 23 March 2022). GSE52917 was the only dataset with a suitable number of samples for each class (sALS and control).

The classification was applied considering: (i) the expression levels of all 101 miRNAs together, (ii) single miRNAs, and (iii) pairwise combinations of miRNAs.

We found an accuracy of 0.58, a sensitivity of 0.66, and a specificity of 0.50 when the classifier was performed on all 101 miRNAs together.

We improved the performance when the classifier considered some single miRNAs (Table 2). We obtained a better classification for miR-139-5p, miR-193b, miR-193b-1, miR-338-5p, miR-455-3p, miR-3911, miR-3911-1, miR-4687-5p, miR-4745-5p, and miR-4763-3p (cutoff of sensitivity > 60% and specificity > 60%). Specifically, miR-338-5p, miR-4745-5p, and miR-4763-3p achieved better performance.

**TABLE 2:** The table shows miRNAs that obtained a better performance (accuracy, sensitivity, and specificity).

miRNA	Accuracy	Sensitivity	Specificity
Hsa-miR-193b	0.66	0.66	0.66
Hsa-miR-3911	0.66	0.66	0.66
Hsa-miR-139-5p	0.66	0.66	0.66
Hsa-miR-193b-1	0.66	0.66	0.66
Hsa-miR-338-5p	0.83	0.66	1
Hsa-miR-3911-1	0.66	0.66	0.66
Hsa-miR-455-3p	0.66	0.66	0.66
Hsa-miR-4687-5p	0.66	0.66	0.66
Hsa-miR-4745-5p	0.83	0.83	0.83
Hsa-miR-4763-3p	0.75	0.66	0.83

*The classification was applied on GSE52917 dataset containing 18 sALS patients and 17 controls.*

Subsequently, we selected those miRNAs of Table 2 and we calculated Accuracy, Sensitivity and Specificity of classification using every possible pairwise combination of miRNAs. As shown, the best classification performance was achieved by miR-4745-5p (Accuracy = 0.83, Sensitivity = 0.83, Specificity = 0.83), although other miRNAs, such as miR-338-5p or miR-4763-3p, reached good classification performances. The Table 3 shows the pairwise miRNAs that obtained the better performance based on the condition of sensitivity and specificity > 70%. The analysis suggests the combination of miR-193b and miR-4745-5p expression could become a potential diagnostic tool, with an accuracy of 0.91, sensitivity of 0.83 and specificity of 1.

**TABLE 3:** The table shows pairwise combinations of miRNAs that achieved a better performance on GSE52917 dataset.

miRNA 1	miRNA 2	Accuracy	Sensitivity	Specificity
Hsa-miR-193b	Hsa-miR-4745-5p	0.91	0.83	1
Hsa-miR-3911	Hsa-miR-4745-5p	0.83	0.83	0.83
Hsa-miR-139-5p	Hsa-miR-4745-5p	0.83	0.83	0.83
Hsa-miR-193b-1	Hsa-miR-4745-5p	0.91	0.83	1
Hsa-miR-338-5p	Hsa-miR-4745-5p	0.83	0.83	0.83
Hsa-miR-3911-1	Hsa-miR-4745-5p	0.83	0.83	0.83
Hsa-miR-4745-5p	Hsa-miR-4763-3p	0.83	0.83	0.83

In summary, the classification analysis on single miRNA suggested 10 miRNAs (miR-139-5p, miR-193b-1, miR-193b, miR-338-5p, miR-455-3p, miR-3911, miR-3911-1, miR-4687-5p, miR-4745-5p, and miR-4763-3p) as potential diagnostic biomarkers of sALS. In addition, we also suggested the combined use of miR-193b and miR-4745-5p expression level evaluation as a sALS diagnostic tool.

## Discussion

The analysis of the literature identified 162 miRNAs, with diagnostic properties, but revealed also several discrepancies among the approaches, the bioptic starting material and the obtained results. Until now, there is no agreement between authors on a diagnostic miRNA profile for sALS. The main limit of these studies are the lack of uniformity among the researchers, the reduced number of individuals included in the study, and the possible differences in ALS-associated miRNA profiles due to the tissues used for the analyses, as also other studies reported [i.e., (2)]. Therefore, the results should be carefully examined. Although extremely useful, the *in vitro* studies may be affected by the culturing conditions. Additionally, the type of cell studied (neuron, astrocytes or glia) or the specific brain area used for miRNA profiling is relevant in identifying specific miRNAs related to sALS. That’s why we also considered miRNA profiles from blood/serum withdrawn. With the improvement of the technological platforms, it appears clear that miRNAs may represent in the future valuable biomarkers for different neurodegenerative conditions (47). The analysis of specific exosomal or microvesicle-associated miRNAs from CSF and peripheral blood can offer potential, easy-accessible biomarkers also for sALS, although a common method for microvesicle or exosome isolation is required as well as a uniform method for the normalization of the analysis. In fact, the alteration of the brain tissue observed in sALS may in turn influence the nature of exosomal and microvesicle miRNA released by neural cells, suggesting that secreted miRNAs may be considered valuable early diagnostic markers for neurodegenerative disease.

The results of the literature analysis revealed a group of 162 possible miRNAs to be used as diagnostic molecules. In order to identify a smaller panel of miRNA, we applied a bioinformatics approach based on the capacity of miRNA to classify sALS from normal subjects. The results of classification performance, made on single miRNA, on an independent dataset suggested 10 miRNAs (miR-139-5p, miR-193b-1, miR-193b, miR-338-5p, miR-455-3p, miR-3911, miR-3911-1, miR-4687-5p, miR-4745-5p, and miR-4763-3p) as potential single diagnostic biomarkers of sALS. Some of them, such as miR-451 (48), or miR-338-3p (49), have been already associated to sALS by other studies. Moreover, miR-338 was upregulated in the spinal cord of *SOD1^G93A^* mouse mice model, where it could be involved in the regulation of glycogen accumulation (50). Koval et al. also found upregulation of miR-338 in both mice and rat *SOD1^G93A^* transgenic model spinal cord (51). Also miR-193b has found downregulated in *SOD1^G93A^* mouse model (52). Several miRNAs of our group, such as miR-193 and miR-338 (53) or miR-139-5p (54) has been also found altered in plasma or serum, respectively, of familial ALS patients. Russell et al. also found miR-455 increased in skeletal muscle of familial ASL patients compared to control subjects (55) suggesting the involvement of this miRNA in muscle wasting.

In addition, the analysis performed considering pairwise combinations of miRNAs indicates the combined use of miR-193b and miR-4745-5p as sALS diagnostic tool. miR-193b-3p has been already described to be involved in the control of autophagy in sALS (52), where it controls the expression of tuberous sclerosis 1 (*TSC1*) to regulate mechanistic target of rapamycin complex 1 (mTORC1) activity. Indeed, *in vitro* studies on NSC-34 cells demonstrated that the loss of miR-193b-3p increase the expression of TSC1, leading to mTORC1 inactivation, protective autophagy and cell survival (52). In motoneuron degeneration, autophagy is a key process necessary to eliminate proteins and organelles by lysosomes, and is recognized as a key decision process for neuronal survival or death (56). The downregulation of miR-193b would be implicated in sALS to promote the death of motoneurons in both NSC-34 cell lines and SOD1*^G93A^* mouse model (52). On miR-4745-5p, it has been found downregulated in sALS (57), although its functional role is still not clear. miR-4745-5p has been found downregulated also in serum of fALS patients (29).

## Future Perspectives and Conclusion

The improvement of technological platform as well as the identification of a gold standard method for the analysis of miRNA profile combined with the bioavailability and stability of miRNAs in biofluids, and the clear definition of the patient cohort will be helpful for the development of a reliable miRNA-based approach for the diagnosis of sALS.

In the view of personalized medicine, we proposed a panel of 10 miRNAs, which are able to correctly classify sALS patients from normal subjects. Among these, the analysis of expression levels of the couple miR-193b/miR-4745-5p in blood, being able to distinguish sALS from healthy subjects, could be translated into clinical practice, in parallel to routine analysis.

## Data Availability Statement

Publicly available datasets were analyzed in this study. This data can be found here: Gene Expression Omnibus, GSE52917.

## Author Contributions

AP and GB analyzed the literature. CC and SD’A performed the *in silico* analyses. All authors contributed to the design of the manuscript and discuss the results altogether.

## Conflict of Interest

The authors declare that the research was conducted in the absence of any commercial or financial relationships that could be construed as a potential conflict of interest.

## Publisher’s Note

All claims expressed in this article are solely those of the authors and do not necessarily represent those of their affiliated organizations, or those of the publisher, the editors and the reviewers. Any product that may be evaluated in this article, or claim that may be made by its manufacturer, is not guaranteed or endorsed by the publisher.
